# Epidemiology of smoking in the rural area of a medium-sized city in Southern Brazil

**DOI:** 10.11606/S1518-8787.2018052000269

**Published:** 2018-09-13

**Authors:** Mariana Otero Xavier, Bianca Del-Ponte, Iná S Santos

**Affiliations:** I Universidade Federal de Pelotas. Faculdade de Medicina. Programa de Pós-Graduação em Epidemiologia. Pelotas, RS, Brasil; II Universidade Federal de Pelotas. Faculdade de Medicina. Programa de Pós-Graduação em Epidemiologia. Departamento de Medicina Social. Pelotas, RS, Brasil

**Keywords:** Adult, Tobacco Use Disorder, epidemiology, Risk Factors, Socioeconomic Factors, Rural Population, Adulto, Tabagismo, epidemiologia, Fatores de Risco, Fatores Socioeconômicos, População Rural

## Abstract

**OBJECTIVE:**

To estimate the prevalence of smoking and associated factors among rural residents.

**METHODS:**

This is a population-based, cross-sectional study of 1,519 individuals carried out in 2016. We randomly selected 24 of the 50 census tracts that make up the eight rural districts of the city of Pelotas, State of Rio Grande do Sul, Brazil. All individuals aged 18 years or more living in the randomly selected households were eligible. Smokers were all those who smoked ≥ 1 cigarette/day for at least one month or declared that they had stopped smoking for less than one month. The independent variables included socioeconomic, demographic, behavioral, and health characteristics. We investigated age of onset, duration of addiction, number of cigarettes smoked/day, pack-years, and types of cigarettes consumed. Poisson regression was performed to calculate the adjusted prevalence ratios (PR) and 95% confidence intervals (95%CI).

**RESULTS:**

The prevalence of smoking was 16.6% (95%CI 13.6–20.0), and it was twice as high in men in relation to women (PR = 1.99, 95%CI 1.44–2.74), in socioeconomic class D or E in relation to class A or B (PR = 2.23, 95%CI 1.37–3.62), and in those who considered their health poor or very poor in relation those with good or very good health (PR = 2.02, 95%CI 1.33–3.08). It was also higher in persons aged 30–59 years (compared to those aged < 30 years), with 5–8 years of education level (compared to those with ≥ 9 years), and with positive screening for alcohol-related disorder. Prevalence was lower among individuals who were overweight or obese than in those with normal weight. Smoking began on average at 16.9 years, with an average consumption of approximately 14 cigarettes/day and mean pack-years of 22 packs/year. The paper hand-rolled cigarette was the most consumed (57.6%).

**CONCLUSIONS:**

Approximately one in six adults in rural Pelotas is a current smoker. The findings show the existence of social inequalities related to smoking addiction. Actions to prevent and control smoking should continue to be stimulated, especially in the most vulnerable subgroups.

## RESUMO

**OBJETIVO:**

Estimar a prevalência de tabagismo e fatores associados entre moradores de zona rural.

**MÉTODOS:**

Estudo transversal de base populacional realizado com 1.519 indivíduos, em 2016. Foram aleatoriamente selecionados 24 dos 50 setores censitários que compõem os oito distritos rurais do município de Pelotas, RS. Indivíduos ≥ 18 anos residentes nos domicílios aleatoriamente selecionados eram elegíveis. Foram considerados tabagistas aqueles que fumavam ≥ 1 cigarro/dia há pelo menos um mês ou que declararam haver parado de fumar há menos de um mês. As variáveis independentes incluíram características socioeconômicas, demográficas, comportamentais e de saúde. Foram investigados: idade de início, duração da adição, número de cigarros fumados/dia, carga tabágica e tipos de cigarros consumidos. Foi realizada regressão de Poisson para cálculo das razões de prevalências (RP) ajustadas e intervalos de confiança de 95% (IC95%).

**RESULTADOS:**

A prevalência de tabagismo foi 16,6% (IC95% 13,6–20,0), sendo duas vezes maior nos homens do que nas mulheres (RP = 1,99; IC95% 1,44–2,74), na classe econômica D ou E do que na A ou B (RP = 2,23; IC95% 1,37–3,62) e naqueles que consideraram sua saúde ruim ou muito ruim, comparados àqueles que a consideraram muito boa ou boa (RP = 2,02; IC95% 1,33–3,08). Também foi superior em pessoas com 30–59 anos (comparadas àquelas com < 30 anos), com 5–8 anos de escolaridade (comparados às pessoas com ≥ 9 anos), e com rastreio positivo para transtorno relacionado ao consumo de álcool. A prevalência foi menor entre indivíduos com sobrepeso ou obesidade, em comparação aos eutróficos. O tabagismo iniciou em média aos 16,9 anos, com consumo médio de cerca de 14 cigarros/dia e carga tabágica média de 22 maços/ano. O cigarro de papel enrolado à mão foi o mais consumido (57,6%).

**CONCLUSÕES:**

Aproximadamente um em cada seis adultos da zona rural de Pelotas é fumante atual. Os achados evidenciam a existência de desigualdades sociais relacionadas à adição tabágica. Ações de prevenção e controle do tabagismo devem continuar sendo estimuladas, sobretudo nos subgrupos mais vulneráveis.

## INTRODUCTION

Smoking is recognized as an epidemic disease, a major risk factor for chronic non-communicable diseases (NCD), and it accounts for approximately six million deaths per year worldwide according to the World Health Organization (WHO)^1^. It is estimated that there are approximately one billion smokers worldwide^1^; in this sense, the goal of the Global Action Plan for the Prevention and Control of NCD is to reduce in 30% the prevalence of smoking between 2013 and 2020^2^.

After recognizing smoking as an important public health problem, Brazil created the National Tobacco Control Program in the 1980s and it signed the Framework Convention on Tobacco Control in 2005 – the first public health treaty in the world, negotiated by the WHO, which has adopted resolutions to curb the global demand for tobacco^3^ ^,^ ^4^. Thus, smoking was banned from closed public places and public transport, tobacco taxes were increased, and cigarette smoking warnings were included in the packs, among other monitoring and prevention measures^3^ ^,^ ^5^.

Brazil has stood out for the adoption of policies to reduce smoking. Among the countries participating in the Global Adult Tobacco Survey (GATS) – a global system for the monitoring of tobacco consumption that uses national surveys with comparable methodologies to obtain information from low- and middle-income countries –, Brazil presented the lowest prevalence of current smokers^6^. National surveys carried out between 1989 and 2013 indicate a continuous decrease in the prevalence of smoking^4^ ^,^ ^5^. Despite this decrease, the prevalence is still high (14.7%) and varies according to sociodemographic characteristics, region of the country, and location (urban or rural area)^5^.

In 2013, the National Health Survey (PNS) evaluated individuals aged 18 years or more from all over Brazil. Smoking was more frequent in the South region and in rural locations^7^. Higher prevalence of smoking in Brazilian rural areas, when compared to urban areas, had already been verified in other studies^3^ ^,^ ^4^. However, we found no population-based studies on behavioral and health factors associated with smoking in rural areas in the country in the literature.

Considering the importance of continuous monitoring to control tobacco consumption, as well as the identification of risk groups for the formulation of qualified care policies, aimed at the particular needs of the population, this study had the objective of estimating the prevalence of smoking and its relation with socioeconomic, demographic, behavioral, and health factors among adults living in a rural area.

## METHODS

This population-based, cross-sectional study was carried out between January and June 2016, with individuals living in the rural area of Pelotas. This city is located in the south of Rio Grande do Sul, Brazil, and has approximately 344,000 inhabitants. Of these, 7% live in the rural area, which has eight districts^8^.

To calculate sample size, we considered a prevalence of smoking of approximately 20%^7^ ^,^ ^9^, 95% confidence level, margin of error of three percentage points, and effect of delineation of 2.0. We added 10% for losses or refusals to the value obtained. The minimum sample required would be 1,458 individuals. For analysis of association, the largest sample size required was 1,737 individuals (to identify association with race), after an increase of 10% for losses or refusals and 15% for the control of confounding. The sampling process was carried out in two stages: first, the primary sample units were 24 randomly selected census tracts, proportional to the number of permanent households in each of the eight district, among the 50 tracts that make up the rural area^8^; then, we identified the community centers of each tract to select the households (30 in each tract). More details on the methodology of the study are available in another publication^10^. We considered eligible all residents aged ≥ 18 years in the households selected.

The outcome of interest was current smoking, and we considered current smokers those who smoked one or more cigarettes per day for at least a month or those who reported having quit smoking for less than one month. Former smokers were those who had stopped smoking for more than one month. The following independent variables were investigated: age of onset and duration of smoking, intensity (average number of cigarettes smoked/day, currently, and throughout the smoking period), periods of interruption, and types of cigarettes consumed currently and throughout the period. The variable of pack-years was constructed using the formula:

[(Current age-age of onset)-(time without smoking)]×mean number of cigarettes/day throughout the smoking period/20

We collected information on the socioeconomic, demographic, behavioral, and health characteristics potentially associated with the outcome^5,11–17^. The socioeconomic variables included the following data: socioeconomic class according to the Brazilian Association of Research Companies (ABEP)^18^ [which considers the possession of goods (television, radio, car, washing machine, VCR or DVD, refrigerator, and freezer) education level of the head of the family, number of toilets/bathrooms in the household, and presence of monthly maid], later categorized into A or B, C, D or E; education level (0–4; 5–8; ≥ 9 full years of study); and, current work situation (no work; no rural work; rural work), which was constructed from questions about current work (yes; no), place of work (city; rural area; both), and the question: “Do you do any rural work, such as those related to planting, animal breeding, fishing, among others? (yes or no)”.

The following demographic characteristics were collected: sex, age (18–29; 30–39; 40–49; 50–59; ≥ 60 years), self-reported race (white; non-white), and marital status (married or with partner; divorced or separated; single; widow/widower).

The behavioral variables were alcohol-related disorder and practice of physical activity. To track the alcohol-related disorder, we used the Alcohol Use Disorder Identification Test (AUDIT)^19^ and we considered positive screening when the individual score ≥ 8 points^20^. The Global Physical Activity Questionnaire (QPAQ)^21^ was used to evaluate the practice of physical activity. This instrument considers physically active the individual with ≥ 150 minutes/week of moderate to vigorous activity in at least one of the three domains – work, transport, and leisure.

The variables related to health were nutritional status, presence of depressive symptoms, and self-perception of health. In order to calculate the nutritional status, we collected weight, using electronic scales (maximum capacity of 150 kilograms and accuracy of 100 grams), and height, using a mountable anthropometer (with a scale of 100 centimeters and graduation in millimeters). We calculated body mass index (BMI) and categorized it as malnutrition (< 18.5 kg/m^2^), eutrophy (18.5–24.9 kg/m^2^), overweight (25–29.9 kg/m^2^), or obesity (≥ 30 kg/m^2^), according to the criteria of the WHO^22^. As the proportion of malnourished individuals was very low (n = 25; 1.7%), they were added to the eutrophic group. The presence of depressive symptoms was evaluated using the Edinburgh Postnatal Depression Scale (EPDS)^23^, considered positive the score ≥ 8 points^24^. Self-perception of health had three categories: very good or good, fair, and poor or very poor.

Data collection was performed by trained and standardized interviewers. We excluded individuals with cognitive or mental disability, who did not had caregivers or relatives; those who were hospitalized or institutionalized during data collection; and, those who did not speak or did not understand Portuguese (a small part of the population was Pomeranian). Losses were those who were not found after at least three attempts of contact, at different days and times, and refusals were those who did not agree to participate in the study. The quality of the data collected was verified by the supervisors of the field work by calling approximately 10% of the sample, which was randomly selected, for the application of a reduced version of the questionnaire. The Kappa coefficient of the variable of smoking (yes; no) was 0.96.

We analyzed the data using the software Stata, version 14.0. For analysis, the outcome was dichotomized into non-smokers, encompassing individuals who never smoked and former smokers, and current smokers. The categorical variables were described as proportions with their respective 95% confidence intervals (95%CI). We calculated the means and medians for the numerical variables and the standard deviation (SD) and interquartile ranges as measures of dispersion.

Initially, we analyzed the prevalence of current smokers, according to the independent variables using the chi-square test of heterogeneity. We performed Poisson regression to obtain the crude and adjusted prevalence ratios (PR) and their respective 95%CI. In the adjusted analysis, we constructed a hierarchical model with three levels. The first (more distal) level included the socioeconomic variables; the second level included the demographic variables; the third level included the behavioral and health variables. The variables were adjusted for the same level and the upper level. All variables were included in the adjusted analysis using backward selection, and we kept those with p-value < 0.20. We used the *svy* command to consider the effect of sample design. Data were weighted according to the number of households sampled in relation to the total number of permanent households in each district.

The project was approved by the Research Ethics Committee of the Faculdade de Medicina of the Universidade Federal de Pelotas (Process 1.363.979). Participation of the individuals was voluntary and the informed consent was read and signed by the participants before data collection.

## RESULTS

We identified 1,697 individuals eligible for the study. Losses accounted for 5.1% (n = 87) and refusals for 5.4% (n = 91) of the total, and 1,519 individuals were interviewed. Losses and refusals were higher among men (p < 0.001) and those aged 18–24 years (p = 0.007).

The prevalence of current smokers and former smokers was 16.6% (95%CI 13.6–20.0) and 18.8% (95%CI 16.7–21.0), respectively. Table 1 describes the sample and prevalence of smoking according to socioeconomic, demographic, behavioral, and health variables. Most individuals belonged to the socioeconomic class C (53.7%), most had a maximum of four years of study (38.7%), and 40.5% were not working. One quarter (26.8%) of the sample was ≥ 60 years old, and most were female (51.7%), white (85.1%), and married or living with a partner (60.3%). Regarding behavioral characteristics, 8.6% presented positive screening for alcohol-related disorder and 83.6% were physically active. Regarding health variables, more than one third were overweight (35.4%) and had positive screening for depressive symptoms (35.3%), and approximately two thirds (64.1%) considered their health very good or good.

**Table 1:** Description of the sample and prevalence of smoking according to socioeconomic, demographic, behavioral, and health variables. Rural area of Pelotas, State of Rio Grande do Sul, Brazil, 2016.

Characteristic	n (%)	Smoking prevalence n (%)	95%CI	p*
Socioeconomic level (ABEP) (n = 1,503)				< 0.001
A or B	301 (20.0)	31 (10.2)	7.0–14.6	
C	814 (53.7)	113 (14.1)	10.9–18.1	
D or E	388 (26.3)	98 (25.7)	19.4–33.2	
Education level (n = 1,509)				0.002
0–4 years	582 (38.7)	106 (18.6)	15.0–22.8	
5–8 years	558 (36.9)	102 (18.5)	14.1–23.8	
≥ 9 years	369 (24.4)	38 (10.7)	7.6–14.9	
Current work situation (n = 1,517)				0.358
No work	613 (40.5)	99 (16.6)	12.9–21.2	
No rural work	395 (26.1)	72 (18.3)	14.8–22.6	
Rural work	509 (33.4)	76 (15.2)	11.8–19.3	
Sex (n = 1,519)				< 0.001
Male	734 (48.3)	159 (21.9)	18.5–25.6	
Female	785 (51.7)	88 (11.6)	8.2–16.2	
Age (years) (n = 1,519)				< 0.001
18–29	287 (18.9)	34 (12.4)	7.3–20.2	
30–39	228 (15.1)	41 (18.4)	13.2–24.9	
40–49	296 (19.6)	60 (20.4)	15.5–26.3	
50–59	297 (19.5)	66 (22.5)	18.1–27.5	
60 or more	411 (26.8)	46 (11.3)	9.0–14.2	
Race (n = 1,519)				0.026
White	1,296 (85.1)	195 (15.2)	12.7–18.2	
Non-white	223 (14.9)	52 (24.0)	16.1–34.3	
Marital status (n = 1,519)				0.012
Married or living with a partner	920 (60.3)	131 (14.4)	11.8–17.5	
Divorced or separated	67 (4.4)	16 (24.0)	15.4–35.3	
Single	397 (26.4)	83 (21.4)	15.7–28.4	
Widow/Widower	135 (8.9)	17 (12.9)	8.1–19.8	
Alcohol consumption (AUDIT) (n = 1,519)				< 0.001
Low risk	1,390 (91.4)	204 (15.0)	12.0–18.6	
Alcohol-related disorder	129 (8.6)	43 (33.5)	25.7–42.2	
Practice of physical activity (GPAQ) (n = 1,441)				0.371
Insufficiently active	235 (16.4)	33 (14.4)	10.0–20.4	
Active	1,206 (83.6)	203 (17.1)	13.8–21.0	
Nutritional status (n = 1,433)				< 0.001
Eutrophy	499 (35.1)	131 (26.5)	20.2–33.8	
Overweight	509 (35.4)	62 (12.5)	9.4–16.5	
Obesity	425 (29.5)	44 (10.5)	6.9–15.6	
Depressive symptoms (EPDS) (n = 1,453)				0.749
No	941 (64.7)	152 (16.5)	12.9–20.9	
Yes	512 (35.3)	88 (17.4)	13.5–22.1	
Self-perception of health (n = 1,507)				0.001
Very good or good	964 (64.1)	142 (14.9)	11.9–18.5	
Fair	461 (30.4)	80 (17.6)	14.9–20.6	
Poor or very poor	82 (5.5)	23 (29.1)	19.6–40.9	
***				***
Total	1,519 (100)	247 (16.6)	13.6–20.0	

ABEP: *Associação Brasileira de Empresas de Pesquisa* (Brazilian Association of Research Companies); AUDIT: Alcohol Use Disorder Identification Test; GPAQ: Global Physical Activity Questionnaire; EPDS: Edinburgh Postnatal Depression Scale

* Chi-square test of heterogeneity.

The prevalence of smoking was higher among persons who were in socioeconomic class D or E, those less educated, men, those aged 50–59 years, non-whites, and those divorced or separated. It was also higher among individuals who were eutrophic, those who perceived health as being poor or very poor, and those with positive screening for alcohol-related disorder, one-third of whom were smokers (Table 1).

The characteristics of the smokers are described in Table 2. Mean age of onset, duration, and current intensity of cigarette consumption were 16.9 years (SD = 5.3), 28.6 years (SD = 14.0), and 14.4 cigarettes/day (SD = 10.8), respectively. Mean pack-years was 22.0 packs/year (SD = 18.7). Two-thirds of the smokers reported having stopped smoking at least once in their lifetime, and mean total abstinence time was 1.1 years (SD = 2.4) (data not shown in the table). The types of cigarettes consumed are shown in the Figure. The most consumed cigarette, throughout the smoking period, was the paper hand-rolled one (57.6%), followed by the industrialized cigarette with filter (39.8%).

**Table 2:** Characteristics of current smokers in the rural area of Pelotas. Pelotas, State of Rio Grande do Sul, Brazil, 2016.

Variable	Mean	Standard deviation	Median	IQR (75-25)
Age of onset (years)	16.9	5.3	16.0	4.0
Duration of smoking (years)*	28.6	14.0	28.0	20.5
Mean current intensity (cigarettes/day)	14.4	10.8	11.0	14.0
Mean lifetime intensity (cigarettes/day)	14.9	9.2	15.0	11.0
18–29 years	12.5	6.8	15.0	15.0
30–39 years	15.0	9.4	15.0	10.0
40–49 years	15.6	9.7	15.0	10.0
50–59 years	15.9	10.8	15.0	11.0
60 years or more	14.0	7.2	12.0	10.0
Pack-years	22.0	18.7	16.2	22.0
18–29 years	5.5	4.0	4.0	5.2
30–39 years	13.8	11.1	11.5	9.1
40–49 years	21.1	15.1	18.2	17.5
50–59 years	29.9	22.4	23.0	28.1
60 years or more	32.9	16.9	32.9	27.1

IQR: interquartile range

* Total smoking time excluding periods of interruption (if they occurred).

^a^ Types of cigarettes currently smoked (each individual could mention more than one type).

^b^ Type of most smoked cigarette throughout life (each individual could only mention one).

**Figure:**
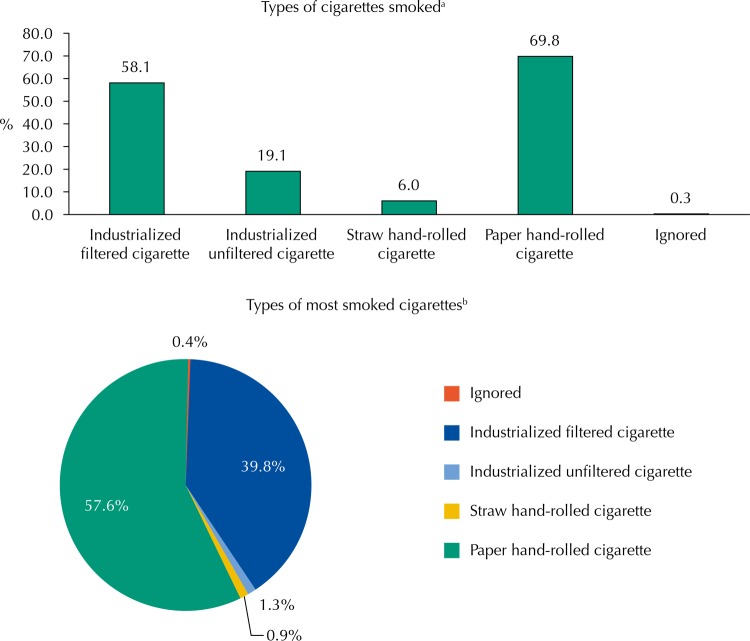
Types of cigarettes smoked by current smokers living in the rural area of Pelotas, State of Rio Grande do Sul, Brazil, 2016.

Table 3 shows the crude and adjusted PR of smoking according to the independent variables. After adjustment, the prevalence of smoking was more than twice as high among those in socioeconomic class D or E (PR = 2.23, 95%CI 1.37–3.62), compared to those in class A or B, and it was 60% higher in those with 5–8 years of education, compared to those with nine years or more (95%CI 1.11–2.42). Prevalence was almost twice as high among men (PR = 1.99, 95%CI 1.44–2.74) and among those aged 50–59 years, compared to those aged 18–29 years (PR = 1.97, 95%CI 1.16–3.35).

**Table 3:** Crude and adjusted analysis of the association between current smokers and socioeconomic, demographic, behavioral, and health variables in the rural area of Pelotas. Pelotas, State of Rio Grande do Sul, Brazil, 2016.

Variable^a^	Current smokers			

	Crude analysis		Adjusted analysis	
	
	PR (95%CI)	p^b^	PR (95%CI)	p^b^
1st level				

Socioeconomic level (ABEP)		< 0.001^c^		0.002^c^
A or B	1		1	
C	1.38 (0.87–2.20)		1.27 (0.80–2.03)	
D or E	2.52 (1.58–4.02)		2.23 (1.37–3.62)	
Education level (full years)		0.004		0.050
0–4	1.74 (1.28–2.37)		1.51 (1.00–2.28)	
5–8	1.73 (1.23–2.44)		1.64 (1.11–2.42)	
≥ 9	1		1	
Current work situation		0.375		0.163
No work	1		1	
No rural work	1.11 (0.83–1.47)		1.28 (0.92–1.79)	
Rural work	0.91 (0.74–1.13)		0.95 (0.78–1.15)	

2nd level				

Sex		< 0.001		< 0.001
Male	1.88 (1.39–2.54)		1.99 (1.44–2.74)	
Female	1		1	
Age (years)		< 0.001		< 0.001
18–29	1		1	
30–39	1.48 (1.01–2.16)		1.62 (1.11–2.37)	
40–49	1.64 (1.06–2.54)		1.84 (1.13–2.99)	
50–59	1.81 (1.12–2.92)		1.97 (1.16–3.35)	
60 or over	0.91 (0.53–1.57)		0.92 (0.52–1.63)	
Race		0.022		0.111
White	1		1	
Non-white	1.58 (1.08–2.31)		1.33 (0.93–1.89)	
Marital status		< 0.001		0.082
Married or living with a partner	1		1	
Divorced or separated	1.66 (1.08–2.56)		1.38 (0.85–2.23)	
Single	1.49 (1.09–2.02)		1.38 (1.03–1.86)	
Widow/ widower	0.89 (0.54–1.49)		1.00 (0.55–1.81)	

3rd level				

Alcohol consumption (AUDIT)		< 0.001		0.037
Low risk	1		1	
Alcohol-related disorder	2.24 (1.60–3.12)		1.47 (1.02–2.10)	
Nutritional status		< 0.001		< 0.001
Eutrophy	1		1	
Overweight	0.47 (0.34–0.65)		0.48 (0.35–0.65)	
Obesity	0.40 (0.24–0.66)		0.41 (0.27–0.63)	
Self-perception of health		0.003		0.008
Very good or good	1		1	
Fair	1.18 (0.97–1.44)		1.15 (0.89–1.48)	
Poor or very poor	1.95 (1.36–2.78)		2.02 (1.33–3.08)	

ABEP: *Associação Brasileira de Empresas de Pesquisa* (Brazilian Association of Research Companies); AUDIT: Alcohol Use Disorder Identification Test

^a^ Variables that remained in the model adjusted for hierarchical levels with p < 0.20.

^b^ Wald test of heterogeneity.

^c^ Wald test for linear trend.

Individuals with positive screening for alcohol-related disorder were approximately 50% more likely to be current smokers, compared to those who had a negative result. Overweight or obese individuals had a lower current prevalence of smoking than eutrophic persons. In addition, smoking was twice as frequent among those who considered their health poor or very poor, compared to those who considered it to be very good or good (Table 3).

To verify the presence of bias in the PR toward the unit from the inclusion of former smokers in the comparison group, the analyses were performed again to compare current smokers exclusively with those who never smoked, which resulted in little variation in the crude and adjusted PR. As losses and refusals were higher among men, we also estimated the prevalence of smoking by assuming that all men were smokers, in which overall prevalence of smoking would become 22.7% (95%CI 20.7–24.7). Subsequently, we assumed the opposite, that is, that all men were non-smokers (never smoked or former smokers), and prevalence of smoking would be 15.0% (95%CI 13.3–16.7).

## DISCUSSION

The proportion of smokers found in this study is consistent with findings for rural areas in Brazil. In the PNS (2013), considering only daily smokers, the prevalence in rural areas was 14.0% (95%CI 12.7–15.2), being it 19.1% (95%CI 17.2–20.9) versus 8.6% (95%CI 7.4–9.8) for men and women respectively^7^. In the countries evaluated by the GATS, the highest prevalence rates have been found in the rural areas of Bangladesh (45.1%) and India (38.4%), and the lowest prevalence rates have been found in the rural areas of Mexico (11.0%) and Egypt (19.7%)^11^.

In Pelotas, a population-based survey conducted with adults living in the urban area in 2010 has identified (using the same criteria as this study) a prevalence of smoking of 21.3% (95%CI 19.3–23.3)^9^, which is higher than that found in the rural area. However, we highlight that the difference in the periods evaluated (2010 and 2016) is important, since the same study^9^ has shown that the prevalence in the urban area decreased from 26.6% to 21.3% (a 20% decrease in six years) between 2004 and 2010^9^. Therefore, the prevalence of smoking in 2016 would be close to that observed in our study if the same rate was kept in the urban area.

The mean age of onset of smoking observed corroborates other studies^25^ ^,^ ^26^. The average of approximately 14 cigarettes/day was similar to that observed in a national survey^6^, as well as in a population-based study in the rural area of Morocco^25^. Furthermore, mean pack-years was 22 packs/year, which increased with age because of the longer time of exposure to smoking.

Regarding cigarette types, the most commonly used was the paper hand-rolled cigarette, which is similar to that observed in 2008 (13.8% in the rural area versus 3.6% in the urban area)^3^. Possible explanations for this finding are the lower cost of rolled cigarettes compared to ready-made ones and the fact that the handmade production of cigarettes reflects a custom or tradition more common in rural communities. However, it is important to highlight that this consumption may be even more harmful to health, since studies have found that individuals who smoke straw or paper hand-rolled cigarettes have a higher prevalence of chronic bronchitis^27^ and chronic obstructive pulmonary disease^28^.

It is not only in Brazil that men smoke more than women. In a study that has evaluated the other countries participating in the GATS, all prevalences were higher in men, which could reflect cultural and religious aspects^11^. Another hypothesis for this sex difference in smoking, specifically in Brazil, is that men are more exposed to risk behaviors and, at the same time, they take less care of their health (use fewer health services)^7^.

In this study, smoking was more frequent in individuals of lower socioeconomic level and with intermediate education level. National surveys have already found similar results^4^. Besides Brazil, higher prevalences of smoking have also been observed in rural areas among the poorest^12^ ^,^ ^29^ and less educated^12^ ^,^ ^13^.

Regarding the higher prevalence of smoking observed in median ages, the findings are also in line with those found in the PNS for daily smokers in rural locations. The prevalence in the national survey was higher among persons aged 40–59 years (21.1%, 95%CI 18.7–23.6) and lower among those aged 18–24 years (3.9%, 95%CI 2.6–5.1%)^7^. Smoking prevention measures may be influencing the smoking habits of young persons. However, this study does not rule out the possibility of survival bias to explain the lower prevalence after the age of 60. This is because, given the close relationship between smoking and various morbidities^1^, smokers may have died at an earlier age from tobacco-related diseases. Older persons may also have stopped smoking because of medical advice or tobacco-related symptoms.

The positive association found between smoking and alcohol-related disorder is already well recognized in the literature and confirmed in other surveys in rural areas^12^ ^,^ ^13^. It should be noted that, in addition to alcohol and cigarettes, other risk factors also tend to coexist, such as smoking and physical inactivity^16^. Although there was no association between smoking and physical activity in our analysis, the direction of the association seemed to follow the inverse, that is, more physically active persons smoked more. As the high prevalence of physically active individuals in this study was mainly due to work-related physical activity, we can assume that rural work, which requires physical labor and happens in the open, would increase the disposition to smoke. In this context, we also expected to find higher prevalences of smoking among those who performed rural activities; however, we observed no association between smoking and type of work. In a study that has evaluated data from the National Household Sample Survey, those who performed agricultural or manual work presented higher frequencies of smoking even after adjusting for income^15^.

We observed an inverse relationship between smoking and nutritional status. Nicotine may increase basal metabolic rate. In addition, the released dopamine and serotonin act in the hypothalamus on the regulation of appetite and satiety^14^. In a subanalysis of this study, we found higher prevalences of overweight and obesity among former smokers compared to current smokers (77.1% and 46.5%, respectively; p < 0.001) when evaluating the association between smoking and nutritional status, even after adjusting for age and sex (data not shown). In addition to a decrease in basal metabolism in the absence of nicotine, the consumption of caloric foods may increase as a compensatory mechanism^14^.

We found a higher probability of being a smoker among those who considered their health poor or very poor. The poorer perception of health among smokers may be related to the greater occurrence of morbidities in these individuals, considering the short, medium, and long term effects of smoking^1^. The relationship between smoking and mental morbidities could be explained by the use of nicotine as a way to relieve tension and modulate emotions^17^; however, no association was found between smoking and depressive symptoms in our study.

As a limitation of this study, we highlight the greater losses and refusals among men, which could underestimate the prevalence of smoking. In fact, in the sensitivity analyses, overall prevalence would be higher if all men considered as losses or refusals smoked (22.7%). Because of the cross-sectional nature of the study, there is the possibility of a reverse causality bias for associations between smoking and behavioral and health variables, which can be bidirectional. Another limitation concerns the information obtained, as there is possible underestimation in the case of undesirable behaviors. Furthermore, *post-hoc* analyses showed that the power was 87.8% for the association between race and smoking. However, power was very low for associations with depressive symptoms and current work (6% and 12.9%, respectively). As the households selected were from the largest centers, residents in more isolated areas may have different behaviors, which may result in some selection bias in the sample, whose direction we cannot predict because we do not know the nature of these behaviors.

Among the positive aspects of this study, the following stand out: the fact that this is the first population-based study carried out with adults in the rural area of Pelotas, the low percentage of losses and refusals, the quality control to verify the repeatability of responses, and the profile of tobacco users. Regarding the latter, the information collected may be useful to local health managers to subsidize actions that combat smoking, such as educational interventions aimed at preventing the onset of smoking among young persons. In addition, this study may be the basis of further analysis of trends and continuous monitoring of tobacco use in the region.

Finally, considering the local peculiarity related to the high production of tobacco in the region^30^, as well as the higher prevalence of smoking in the South of the country^5^, the data showed that the prevalence of smoking in the rural area of Pelotas was lower than expected, which is closer to that found in a national survey for the rural areas of Brazil^7^. In addition, the pattern of cigarette consumption in the rural locations of Pelotas reflects the social inequalities in Brazil, since poorer and less educated individuals are more likely to smoke.
